# Effect of Green‐Synthesized Silver Nanoparticles From *Nepeta pogonosperma* and *Astrodaucus persicus* on the Reduction of *Bap* Gene Expression in Strong Biofilm‐Producing *Acinetobacter baumannii* Clinical Isolates

**DOI:** 10.1002/mbo3.70391

**Published:** 2026-08-31

**Authors:** Maedeh Kakavan, Mehrdad Gholami, Mohammad Ahanjan, Mohammad Ali Ebrahimzadeh, Abolfazl Hossein Nataj, Tahoora Mousavi, Hamid Reza Goli

**Affiliations:** ^1^ Immunogenetics Research Centre, Faculty of Medicine Mazandaran University of Medical Sciences Sari Iran; ^2^ Department of Medical Microbiology and Virology, Faculty of Medicine Mazandaran University of Medical Sciences Sari Iran; ^3^ Pharmaceutical Sciences Research Center, School of Pharmacy Mazandaran University of Medical Sciences Sari Iran; ^4^ Department of Biostatistics and Epidemiology, Faculty of Health Mazandaran University of Medical Sciences Sari Iran; ^5^ Molecular and Cell Biology Research Center, Hemoglobinopathy Institute, Faculty of Medicine Mazandaran University of Medical Sciences Sari Iran

**Keywords:** *Acinetobacter baumannii*, *Astrodaucus persicus*, bap, biofilm, green silver nanoparticles, *Nepeta pogonosperma*

## Abstract

Given the critical role of the *bap* gene in biofilm formation of *Acinetobacter baumannii*, this study aimed to evaluate the efficacy of green‐synthesized silver nanoparticles (AgNPs) derived from *Nepeta pogonosperma* and *Astrodaucus persicus* (Boiss) in reducing the expression of this gene and inhibiting biofilm production in clinical isolates of *A. baumannii*. AgNPs were synthesized using the leaf extracts of the plants and characterized using standard techniques. The minimum inhibitory concentration of the AgNPs was determined by the microbroth dilution method. Biofilm production and the anti‐biofilm effects of the nanoparticles were assessed using a microtiter plate assay. Quantitative real‐time PCR was employed to evaluate *bap* gene expression levels before and after treatment with the AgNPs. The AgNPs significantly inhibited biofilm formation in *A. baumannii*. Furthermore, a marked reduction in *bap* gene expression was observed following treatment. Moreover, 90% and 10% of the isolates showed up to 60% and 61%–80% of biofilm disruption after treatment with *A. persicus* AgNP, respectively. In addition, 80%, 15%, and 5% of the isolates showed up to 60%, 61%–80%, and more than 80% of biofilm disruption after treatment with *N. pogonosperma* AgNP, respectively. Green synthesis of silver nanoparticles represent a promising strategy to counteract antimicrobial resistance. The AgNPs investigated in this study demonstrated significant potential in inhibiting biofilm formation and reducing *bap* gene expression in strong biofilm‐producing clinical isolates of *A. baumannii*. Further research is warranted to elucidate the underlying mechanisms and optimize the clinical application of these nanoparticles.

## Graphical abstract

Green‐synthesized silver nanoparticles from *Nepeta pogonosperma* and *Astrodaucus persicus* significantly inhibit biofilm formation and downregulate *bap* gene expression in multidrug‐resistant clinical isolates of *Acinetobacter baumannii*. This eco‐friendly approach offers a promising strategy to combat antimicrobial resistance.

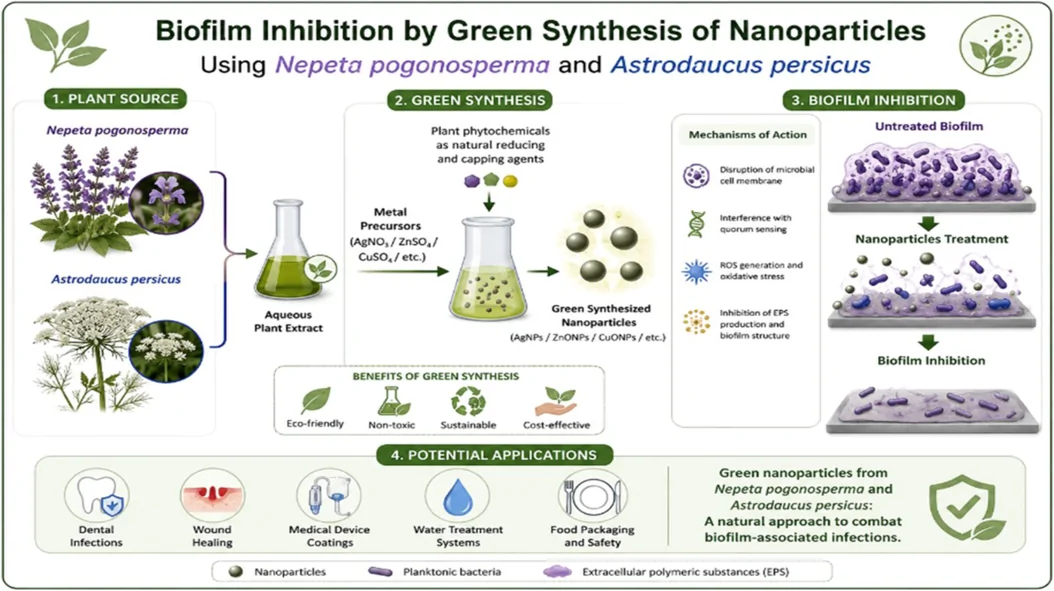

**Abbreviations**
AgNPs: silver nanoparticles
ATCC: American Type Culture Collection
Bap: biofilm‐associated protein
cDNA: complementary DNA
CFU: colony forming unit
CLSI: Clinical and Laboratory Standards Institute
DEPC: diethyl pyrocarbonate
DNA: deoxyribonucleic acid
ELISA: enzyme‐linked immunosorbent assay
ICU: intensive care unit
MBC: minimum bactericidal concentration
MDR: multidrug resistant
MHB: Mueller‐Hinton Broth
MIC: minimum inhibitory concentration
OD: optical density
ODc: optical density cut‐off
PBS: phosphate buffered saline
PCR: polymerase chain reaction
qRT‐PCR: quantitative real‐time PCR
RNA: ribonucleic acid
SPSS: Statistical Package for the Social Sciences.
TSA: tryptic soy agar
TSB: trypticase soy broth

## 1 Introduction

*Acinetobacter baumannii* is a Gram‐negative opportunistic coccobacillus causing nosocomial and community‐acquired infections (Lee et al. 2017). Its resistance to last‐line antibiotics, including colistin, tigecycline, and carbapenems, has classified it among the most problematic hospital pathogens within the ESKAPE group (*Enterococcus faecium*, *Staphylococcus aureus*, *Klebsiella pneumoniae*, *A. baumannii*, *Pseudomonas aeruginosa*, and *Enterobacter spp*.) (Pompilio et al. 2021). The emergence of novel resistance mechanisms and the evolution of existing ones render this organism a persistent global threat to public health and a major complication in treatment regimens (Whiteway et al. 2022; Kyriakidis et al. 2021).

The survival and antimicrobial resistance of *A. baumannii* are closely linked to the ability of clinical strains to form biofilms on biotic or abiotic surfaces (Espinal et al. 2012). Biofilms are encased in a matrix of polysaccharides, proteins, and extracellular DNA. Furthermore, bacteria within a biofilm exhibit gene expression profiles distinct from their planktonic counterparts, resulting in significant alterations in morphology, physicochemical properties, and antibiotic susceptibility (Liu et al. 2020). The common stages of biofilm formation, including initial attachment, microcolony formation, maturation, and dispersal, are regulated by quorum‐sensing (QS) systems (Muhsin et al. 2015). Several virulence factors, including Bap, OmpA, CsuE, EPS, BfmS/BfmR, PER‐1, poly‐N‐ (Lee et al. 2017; Liu et al. 2020)‐β‐acetylglucosamine (PNAG), and quorum sensing, contribute to biofilm formation in *A. baumannii* (Jahangiri et al. 2019). The biofilm‐associated protein (Bap), encoded by the *bap* gene, plays a critical role in biofilm adhesion to bronchial cells, structural integrity, and water channel development. However, disruption of the *bap* gene leads to decreased biofilm thickness, volume, and interbacterial cell adhesion (Shakib et al. 2022).

Biofilm‐associated infections are notoriously difficult to treat, as standard antibiotic regimens often fail to eradicate them due to enhanced resistance and genetic adaptations (Ciofu and Tolker‐Nielsen 2019). The biofilm phenotype confers substantial protection to bacterial cells, promoting persistence through mechanisms such as enzymatic degradation of antimicrobials, efflux pump activity, and reduced permeability (Balcázar et al. 2015). Consequently, several strategies have been developed to inhibit *A. baumannii* biofilm formation and pathogenicity, including the use of synthetic chemicals and naturally derived agents (Roy et al. 2018). High‐efficacy natural anti‐biofilm agents typically function by interfering with specific stages of biofilm development or by silencing QS signaling and suppressing the expression of associated genes (Paul et al. 2019). Plant‐derived compounds such as phenols, polyphenols, terpenoids, essential oils, lectins, polypeptides, and alkaloids possess significant antibacterial properties, and their complex mixtures may exhibit synergistic anti‐biofilm activity (Cowan 1999). Additionally, synthetic antimicrobial compounds can be designed with a specific focus on biofilm inhibition, as their structure‐activity relationships are often more readily defined (Chen et al. 2020).

Nanoparticles have emerged as a promising class of anti‐biofilm and antimicrobial agents to combat microbial infections (Cruz‐Muñiz et al. 2017). The green synthesis of silver nanoparticles (AgNPs) has garnered particular interest due to its cost‐effectiveness and eco‐friendliness, presenting a viable alternative strategy to address antimicrobial resistance (Wintachai et al. 2019). Nanoparticles can disrupt bacterial outer membranes, interfere with ion exchange and enzymatic activity, alter gene expression, and induce cell death pathways. Moreover, they often exhibit greater stability and lower toxicity compared to conventional antibiotics (Zendegani and Dolatabadi 2020). *Nepeta*, a genus comprising approximately 300 species within the *Lamiaceae* family, represents a valuable resource in traditional medicine across India, Pakistan, Nepal, China, Turkey, and Iran. *Nepeta pogonosperma*, identified in 1984 (Jamzad and Assadi 1984) is traditionally used to treat conditions such as chickenpox, tuberculosis, malaria, pneumonia, influenza, measles, stomach disorders, eye complaints, respiratory ailments, asthma, colds, and coughs (Sharma et al. 2021).

The Apiaceae family, one of the largest and most well‐known families of flowering plants, includes 300–450 genera and 3000–3700 species worldwide (Hesari et al. 2021). *Astrodaucus persicus* (Boiss) is a notable medicinal plant within this family, native primarily to Asia and prevalent in Iran, Turkey, Syria, and Iraq. In Iran and Turkey, its aerial parts and young roots are traditionally used as food additives (Bodaghabadi et al. 2021). Plants from the Apiaceae family exhibit diverse biological activities, including vasorelaxant, antibacterial, hepatoprotective, antitumor, and apoptosis‐inducing effects (Hesari et al. 2021).

In the green synthesis of silver nanoparticles, extracts from plants, algae, fungi, and microbes serve as reducing and capping agents. Phytochemicals such as flavonoids, terpenoids, and polysaccharides act as electron donors to reduce Ag^+^ ions while simultaneously stabilizing the formed nanoparticles (Jegadeeswaran et al. 2012). Given the critical role of biofilm production in the antibiotic resistance and pathogenicity of *A. baumannii* clinical isolates, inhibiting biofilm formation represents a potential strategy to reduce mortality associated with this organism. Therefore, this study aimed to investigate the efficacy of silver nanoparticles synthesized from *N. pogonosperma* and *A. persicus* extracts in inhibiting biofilm formation.

## 2 Materials and Methods

### 2.1 Study Design, Bacterial Isolation, and Identification

In this cross‐sectional study, 100 clinical isolates of *Acinetobacter baumannii* (determined based on a 95% confidence level and a 6% margin of error) were collected (Fakhuruddin Indhar et al. 2017). Strains were randomly obtained from wounds, urine, and blood samples at a burn hospital affiliated with Mazandaran University of Medical Sciences in northern Iran. Isolates were identified using conventional microbiological and biochemical methods and confirmed by polymerase chain reaction (PCR) targeting the *bla*_*OXA‐51*_ β‐lactamase encoding gene (Khuntayaporn et al. 2021).

### 2.2 Biofilm Formation Assay

Biofilm formation was assessed in microtiter plates as previously described by Kakavan et al. (2025). Briefly, bacteria were grown in microtiter plate wells, non‐adherent cells were removed by washing, and the remaining biofilm was stained with 0.1% (w/v) crystal violet. The absorbance of the dissolved stain was measured using a microtiter plate reader (Stat Fax 2100) to quantify biofilm formation.

### 2.3 Preparation of Green‐Synthesized Silver Nanoparticles

The experimental protocol was approved by the Ethics Committee of Mazandaran University of Medical Sciences (No. 21223). Plant materials were collected from Kiasar, Mazandaran, Iran, and authenticated by Dr. Bahman Eslami (Assistant Professor of Plant Systematics and Ecology). Voucher specimens (No. 1375‐1376) were deposited in the herbarium of the Sari School of Pharmacy. The AgNPs used in this study were kindly provided by Professor Mohammad Ali Ebrahimzadeh (School of Pharmacy, Mazandaran University of Medical Sciences), synthesized according to a previously published protocol (Ebrahimzadeh et al. 2023).

### 2.4 Determination of Minimum Inhibitory Concentration (MIC)

The MIC of the AgNPs against the 100 clinical isolates was determined using the microbroth dilution method (CLSI 2021). The initial concentrations of *N. pogonosperma* and *A. persicus* AgNPs were 300 µg/mL and 200 µg/mL, respectively. Serial dilutions were performed in Mueller Hinton Broth (MHB; Merck, Germany) in a 96‐well plate. *N. pogonosperma* AgNPs (AgNP@Np) were diluted from 75 to 0.146 µg/mL, and *A. persicus* AgNPs (AgNP@Ap) from 50 to 0.097 µg/mL. A bacterial suspension of 10^5^ CFU/well was added to each well. Positive controls (medium and bacteria) and negative controls (medium and nanoparticles) were included. Plates were incubated at 37°C for 24 h, and the MIC was defined as the lowest concentration showing no visible turbidity (Rezania et al. 2022).

### 2.5 Minimum Bactericidal Concentration (MBC)

The MBC was determined in accordance with Clinical and Laboratory Standards Institute (CLSI) guidelines (CLSI 2021). Briefly, 10 µL of bacterial suspension from wells showing no growth in the MIC assay were plated onto Tryptic Soy Agar (TSA). The MBC was defined as the lowest concentration that resulted in no bacterial growth after 24 h of incubation at 37°C.

### 2.6 Determination of Biofilm Inhibition and Disruption

The effect of AgNPs on biofilm inhibition was evaluated using a microtiter plate assay as described by Kim et al (Kim et al. 2018)., with modifications. Bacterial suspensions (1–2 × 10^6^ CFU/mL) in Tryptic Soy Broth (TSB) were combined with sub‐MIC concentrations of AgNPs in a 96‐well plate and incubated for 24 h at 37°C (final volume 200 µL/well). The required volumes were calculated using the formula C1·V1 = C2·V2, where C1 is the stock AgNP concentration, V1 is the volume of AgNPs, C2 is the desired sub‐MIC concentration, and V2 is the final well volume. The sub‐MIC concentrations used were 1 µg/mL for AgNP@Ap and 1.171 µg/mL for AgNP@Np.

After incubation, the supernatant was carefully aspirated, and the biofilm was fixed with 99% methanol for 15 min. Following methanol removal, biofilms were stained with 2% (w/v) crystal violet for 15 min, washed thoroughly with distilled water, and dissolved in 160 µL of 33% glacial acetic acid. Absorbance was measured at 630 nm using a Stat Fax 2100 microplate reader.

Biofilm disruption was assessed based on a method by Chandrasekharan et al. (2022). with slight modifications. Twenty strong biofilm‐producing isolates were selected based on optical density. Bacterial suspensions (1–2 × 10^6^ CFU/mL) in TSB were added to a 96‐well plate and incubated for 5 h at 37°C to allow initial adhesion. The medium was then replaced with fresh TSB containing the sub‐MIC concentrations of AgNPs, and incubation continued for 19 h. Planktonic cells were removed by washing with saline, and the remaining biofilm was fixed, stained, and quantified as described above. Wells containing untreated cells were used as controls. The percentage of biofilm inhibition or disruption was calculated using the following formula (Shinde et al. 2021):

%Biofilm inhibition/disruption=[OD untreated cells–OD treated cells/OD untreated cells]×100.

### 2.7 Investigation of *Bap* Gene Expression by qRT‐PCR

Total RNA was extracted from the isolates using the YTzol pure RNA solution (Wizbiosolutions), following the manufacturer's instructions, which involved steps with chloroform, isopropanol, and treatment with DNase I (Wizbiosolutions), as previously described (Kakavan et al. 2025). Complementary DNA (cDNA) was synthesized using the RevertAid First Strand cDNA Synthesis Kit (Yekta Tajhiz, Iran). Quantitative real‐time PCR (qRT‐PCR) was performed using specific primers for the *bap* gene (*bap*‐F: 5ʹ‐TAGACGCAATGGATAACG‐3ʹ; *bap*‐R: 5ʹ‐TTAGAACCGATAACGATACC‐3ʹ). The *16S rRNA* gene was used as an endogenous control for normalization (*16S rRNA*‐F: 5´‐TCAGCTCGTGTCGTGAGATG‐3´; *16S rRNA*‐R: 5´‐CGTAAGGGCCATGATG‐3ʹ) (Kakavan et al. 2025).

### 2.8 Real‐Time PCR Following Treatment With Silver Nanoparticles

Strong biofilm‐producing isolates were treated with AgNPs in two separate experiments with durations of 24 and 48 h. Initially, isolates were cultured on blood agar and incubated for 24 h at 37°C. A 0.5 McFarland suspension was prepared in TSB containing AgNPs at sub‐MIC concentrations (1/2 MIC) and incubated for 24 h at 37°C. The bacterial cells were then washed three times with sterile phosphate‐buffered saline (PBS), followed by RNA extraction and cDNA synthesis for qRT‐PCR analysis.

For the 48‐h treatment, a 0.5 McFarland suspension in TSB with sub‐MIC AgNPs was incubated for 48 h at 37°C. Cells were subsequently washed, and RNA extraction and cDNA synthesis were performed prior to qRT‐PCR. The relative change in *bap* gene expression was calculated using the 2^‾∆∆Ct^ method (Livak method) (Livak and Schmittgen 2001), with *A. baumannii* ATCC 15308 used as the reference strain.

### 2.9 Statistical Analysis

Statistical analyses were performed using SPSS version 24. Categorical variables (e.g., biofilm intensity vs*. bap* expression groups) were compared using the Chi‐square test or Fisher's exact test, as appropriate. The Kruskal‐Wallis H test was used to compare continuous non‐parametric data (e.g., fold‐change in gene expression) across biofilm intensity groups. A *p*‐value of < 0.05 was considered statistically significant.

## 3 Results

### 3.1 Isolate Characteristics and Distribution

A total of 100 *A. baumannii* clinical isolates were collected from 50 male and 50 female patients hospitalized at a burn center in northern Iran. The age of the participants ranged from 6 months to 88 years (mean ± SD: 42.08 ± 25.08 years). The isolates were distributed across various hospital departments: adult burn department (34%), intensive care units (29%), surgical sections (21%), and pediatric burn department (16%). The isolates originated from wound samples (73%), urine (15%), and blood (12%).

### 3.2 MIC and MBC of Silver Nanoparticles

The antimicrobial activity of the green‐synthesized AgNPs was evaluated against all isolates. The MIC and MBC values for both types of AgNPs ranged from 0.1 to 40 μg/mL (Table 1).

**Table 1:** Number of isolates with different MIC and MBC values (µg/mL) for the silver nanoparticles.

Silver nanoparticles	Concentration (μg/mL)	MIC	MBC
*Nepeta pogonosperma*	0.1–0.5	52	26
	1–3	43	68
	4–10	4	4
	11–20	1	1
	21–40	—	1
	> 40	—	—
*Astrodaucus persicus*	0.1–0.5	80	61
	1–3	19	37
	4–10	—	—
	11–20	—	1
	21–40	1	1
	> 40	—	—

### 3.3 Inhibition of Biofilm Formation by Silver Nanoparticles

**Figure 1:**
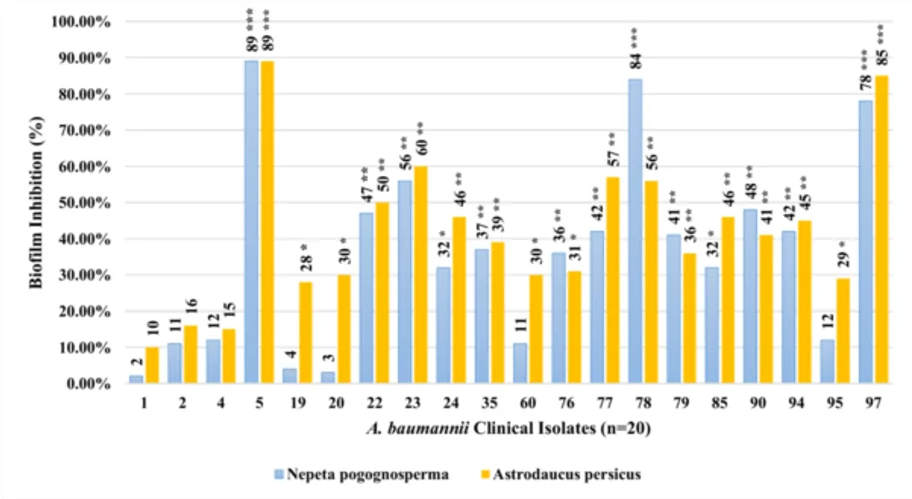
Inhibition of biofilm formation by AgNPs. Biofilm formation by *A. baumannii* clinical isolates (*n* = 20 strong producers) was assessed in the presence of sub‐MIC concentrations of AgNP@Np (1.171 µg/mL) and AgNP@Ap (1 µg/mL) using a microtiter plate crystal violet assay. The percentage reduction in biofilm formation is shown relative to an untreated control. Data are presented as mean ± SD from three independent experiments. *p < 0.05, **p < 0.01, ***p < 0.001 compared to untreated control; #*p* < 0.05 between AgNP@Np and AgNP@Ap treatments.

Treatment with AgNP@Np and AgNP@Ap resulted in varying degrees of biofilm inhibition. For AgNP@Ap: 15% (3/20) of isolates showed up to 20% inhibition; 35% (7/20) showed 21%–40% inhibition; 40% (8/20) showed 41%–60% inhibition; 10% (2/20) showed > 80% inhibition; and no isolates showed 61–80% inhibition. For AgNP@Np: 35% (7/20) showed up to 20% inhibition; 20% (4/20) showed 21%–40% inhibition; 30% (6/20) showed 41%–60% inhibition; 5% (1/20) showed 61%–80% inhibition; and 10% (2/20) showed > 80% inhibition. These results demonstrate the anti‐biofilm potential of both AgNPs (Figure 1).

### 3.4 Disruption of Pre‐Formed Biofilms by Silver Nanoparticles

**Figure 2:**
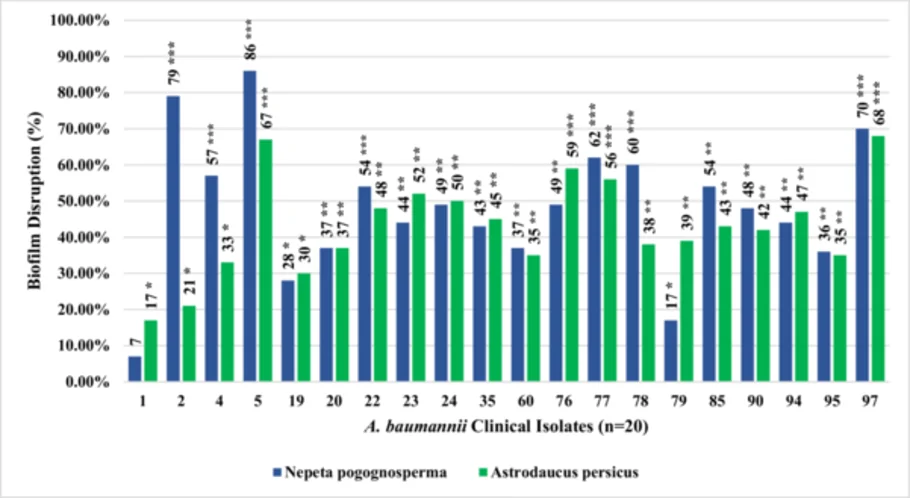
Disruption of **pre‐formed** biofilm by AgNPs. Biofilms formed by A. baumannii clinical isolates (*n* = 20 strong producers) were treated with sub‐MIC concentrations of AgNP@Np (1.171 µg/mL) and AgNP@Ap (1 µg/mL). The percentage reduction in biofilm biomass is shown relative to untreated controls. Data are presented as mean ± SD. *p < 0.05, **p < 0.01, ***p < 0.001 versus untreated control.

The ability of AgNPs to disrupt established biofilms was also evaluated. Following exposure to AgNP@Ap: 5% (1/20) of isolates showed up to 20% disruption; 40% (8/20) showed 21%–40% disruption; 45% (9/20) showed 41%–60% disruption; 10% (2/20) showed 61%–80% disruption; and none showed > 80% disruption. For AgNP@Np: 10% (2/20) showed up to 20% disruption; 20% (4/20) showed 21–40% disruption; 50% (10/20) showed 41%–60% disruption; 15% (3/20) showed 61%–80% disruption; and 5% (1/20) showed > 80% disruption (Figure 2). These findings indicate that both AgNPs can effectively disrupt pre‐formed biofilms, albeit with varying efficacy.

### 3.5 Relationship etween Biofilm Formation Intensity and MIC/MBC of AgNPs

A significant correlation was observed between the intensity of biofilm formation and the MIC/MBC values of the AgNPs from both plants (Chi‐square test, *p* < 0.05). Over 90% of the biofilm‐forming strains, classified as strong or moderate producers, exhibited MIC and MBC values between 0.1 and 3 μg/mL (Table 2).

**Table 2:** Correlation between biofilm formation intensity and MIC/MBC ranges (μg/mL) AgNPs.

Biofim intensity		Weak (*n* = 2)	Moderate (*n* = 49)	Strong (*n* = 49)
MIC & MBC ranges (µg/mL)				
MIC of AgNP@Np	0.1–0.5	2 (100%)	30 (61.22%)	20 (40.81%)
	1–3	0	16 (32.65%)	27 (55.10%)
	4–10	0	2 (4.08%)	2 (4.08%)
	11–20	0	1 (2.04%)	0
MIC of AgNP@Ap	0.1–0.5	2 (100%)	39 (79.59%)	39 (79.59%)
	1–3	0	9 (18.36%)	10 (20.40%)
	21–40	0	1 (2.04%)	0
MBC AgNP@Np	0.1–0.5	0	14 (28.57%)	12 (24.48%)
	1–3	2 (100%)	32 (65.30%)	34 (69.38%)
	4–10	0	2 (4.08%)	2 (4.08%)
	11–20	0	0	1 (2.04%)
	21–40	0	1 (2.04%)	0
MBC AgNP@Ap	0.1–0.5	2 (100%)	31 (63.26%)	28 (57.14%)
	1–3	0	17 (34.69%)	20 (40.81%)
	11–20	0	0	1 (2.04%)
	21–40	0	1 (2.04%)	0

### 3.6 *bap* Gene Expression and Its Correlation With Biofilm Formation

Among the 100 clinical isolates, 93 exhibited elevated *bap* gene expression. The distribution of fold‐changes was as follows: 49.46% (46/93) showed a 2–5 fold increase, of which 44 isolates were moderate biofilm producers; 16.12% (15/93) showed a 6–10 fold increase; 16.12% (15/93) showed an 11–15 fold increase; and 18.27% (17/93) showed a 16–20 fold increase. All isolates in the latter three groups were strong biofilm producers. Seven isolates showed no change in *bap* expression; five were moderate and two were weak biofilm producers. Strains with strong biofilm formation ability demonstrated significantly higher *bap* gene expression levels (Kruskal‐Wallis H test, *p* < 0.05) (Table 3).

**Table 3:** Relationship between *bap* gene expression level and biofilm formation intensity.

Fold Changes	Biofilm intensity		
	Strong (*n* = 49)	Moderate (*n* = 49)	Weak (*n* = 2)
No change	0	5 (10.20%)	2 (100%)
2–5 fold	2 (4.08%)	44 (89.79%)	0
6–10 fold	15 (30.61%)	0	0
11–15 fold	15 (30.61%)	0	0
16–20 fold	17 (34.69%)	0	0

### 3.7 Association Between MIC/MBC of NPs and *Bap* Gene Expression

A significant correlation was found between the level of *bap* gene expression and the MIC/MBC values of the AgNPs (Chi‐square test, *p* < 0.05). Isolates with higher *bap* expression tended to have lower MIC and MBC values for both types of AgNPs, indicating greater susceptibility (Tables 4 and 5).

**Table 4:** Correlation between MIC and MBCof AgNP@Ap and *bap* gene expression.

Fold Changes	MIC of AgNP@Ap *(µg/ml)*					MBC of AgNP@Ap *(µg/ml)*						
	0.1–0.5	1–3	4–10	11–20	20–40	Total	0.1–0.5	1–3	4–10	11–20	20–40	Total
No change	7 (100%)	0	0	0	0	7	5 (71.42%)	2 (28.57%)	0	0	0	7
2–5 fold	36 (78.26%)	9 (19.56%)	0	0	1 (2.17%)	46	29 (63.04%)	16 (34.78%)	0	0	1 (2.17%)	46
6–10 fold	12 (80%)	3 (20%)	0	0	0	15	8 (53.33%)	6 (40%)	0	1 (6.66%)	0	15
11–15 fold	10 (66.6%)	5 (33.33%)	0	0	0	15	7 (46.66%)	8 (53.33%)	0	0	0	15
16–20 fold	15 (88.23%)	2 (11.76%)	0	0	0	17	12 (70.58%)	5 (29.41%)	0	0	0	17

**Table 5:** Correlation between MIC and MBC of AgNP@Np and *bap* gene expression.

Fold changes	MIC of AgNP@Np (µg/mL)					MBC of AgNP@Np (µg/mL)						
	0.1–0.5	1–3	4–10	11–20	20–40	Total	0.1–0.5	1–3	4–10	11–20	20–40	Total
No change	6 (85.71%)	1 (14.28%)	0	0	0	7	2 (28.57%)	5 (71.42%)	0	0	0	7
2–5 fold	28 (60.86%)	15 (32.60%)	2 (4.34%)	1 (2.17%)	0	46	13 (28.26%)	30 (65.21%)	2 (4.34%)	0	1 (2.17%)	46
6–10 fold	6 (40%)	9 (60%)	0	0	0	15	2 (13.33%)	13 (86.66%)	0	0	0	15
11–15 fold	7 (46.66%)	7 (46.66%)	1 (6.66%)	0	0	15	5 (33.33%)	9 (60%)	1 (6.66%)	0	0	15
16–20 fold	5 (29.41%)	11 (64.70%)	1 (5.88%)	0	0	17	4 (23.52%)	11 (64.70%)	1 (5.88%)	1 (5.88%)	0	17

### 3.8 Effect of Silver Nanoparticles on *Bap* Gene Expression

**Figure 3:**
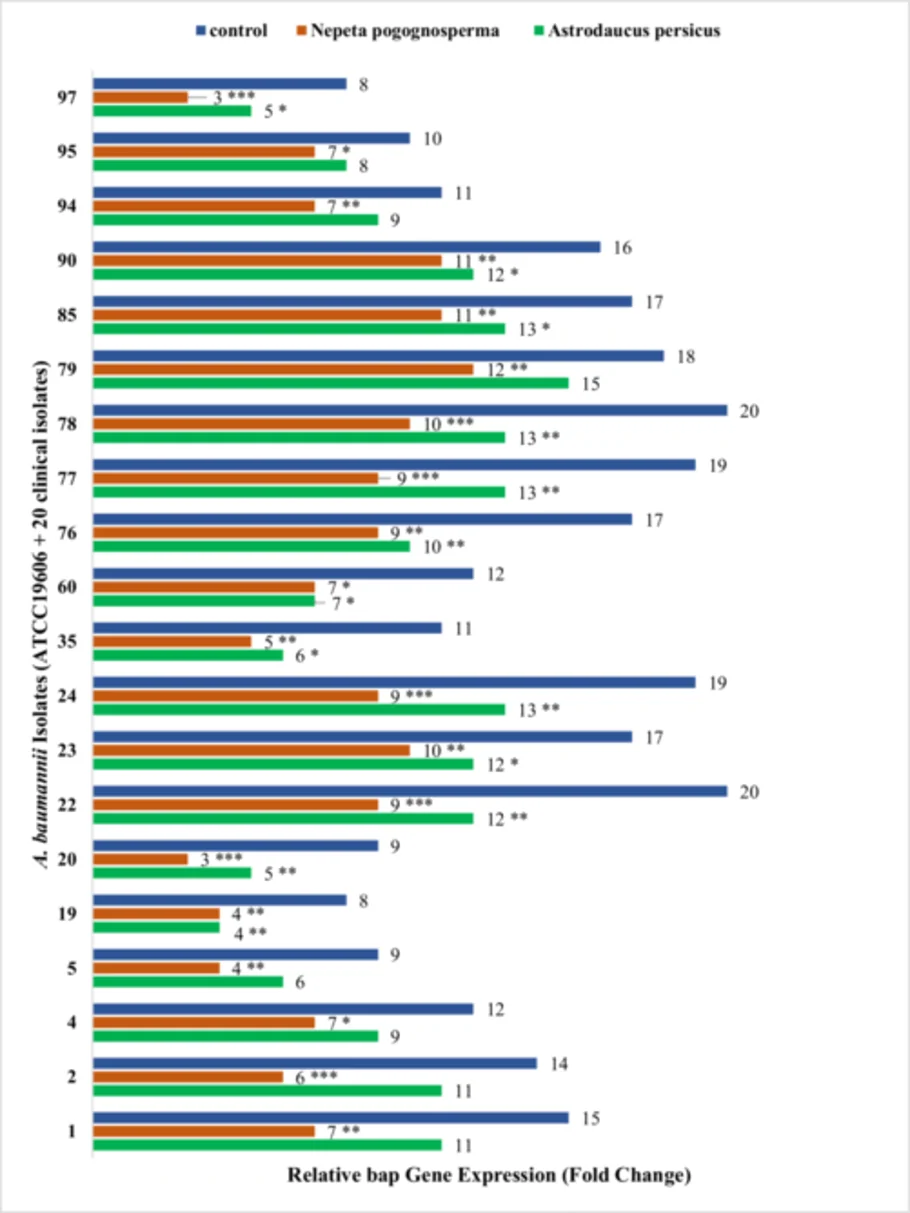
Reduction of *bap* gene expression after 24‐h treatment with AgNPs. Strong biofilm‐producing A. baumannii isolates (*n* = 20) and ATCC19606 were treated with sub‐MIC concentrations of AgNP@Np (1.171 µg/mL) and AgNP@Ap (1 µg/mL) for 24 h. Relative expression was normalized to the 16S rRNA gene and calculated using the 2^‐ΔΔCt method. Data represent mean ± SD from three independent experiments. *p < 0.05, **p < 0.01, ***p < 0.001 versus untreated control.

**Figure 4:**
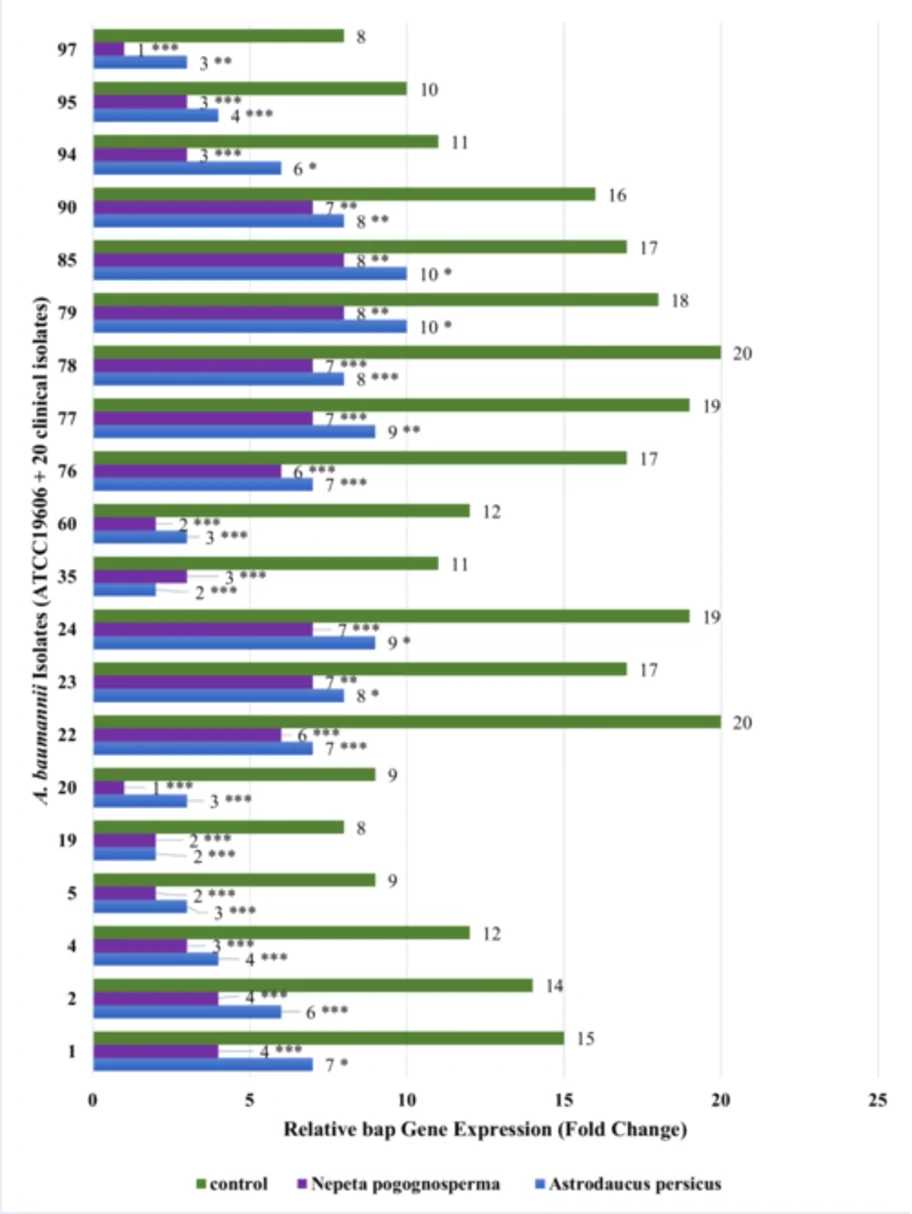
Reduction of *bap* gene expression after 48‐h treatment with AgNPs. Strong biofilm‐producing A. baumannii isolates (*n* = 20) and ATCC19606 were treated with sub‐MIC concentrations of AgNP@Np (1.171 µg/mL) and AgNP@Ap (1 µg/mL) for 48 h. Relative expression was normalized to the 16S rRNA gene. Data are shown as mean ± SD. *p < 0.05, **p < 0.01, ***p < 0.001 versus untreated control.

Treatment with sub‐MIC concentrations of AgNP@Ap (1 μg/mL) and AgNP@Np (1.171 μg/mL) significantly reduced bap gene expression in A. baumannii ATCC19606 and the 20 strong biofilm‐producing isolates compared to the untreated control (*p* < 0.05 for both AgNP types at 24 h; *p* < 0.01 for AgNP@Ap and *p* < 0.001 for AgNP@Np at 48 h). The reduction was more pronounced after 48 h of treatment (*p* < 0.05 compared to 24 h). No significant difference was observed between AgNP@Np and AgNP@Ap in their ability to reduce bap expression at either time point (*p* > 0.05). The *16S rRNA* gene was used as an internal control. Reductions in expression were observed after both 24 and 48 h of treatment (Figures 3 and 4).

## 4 Discussion

*Acinetobacter baumannii* is renowned for its capacity to form biofilms, which act as protective barriers against antibiotics and host immune responses (Nocera et al. 2021). The widespread distribution of biofilms on medical devices, including catheters, ventilators, and implants complicates the management of *A. baumannii* infections more challenging (Cavallo et al. 2023). Understanding the mechanisms of biofilm development is therefore crucial for devising effective countermeasures (Upmanyu et al. 2022). Among the innovative strategies being explored, nanoparticles show significant promise for biofilm disruption and antibiotic potentiation (Mohanta et al. 2023). Green‐synthesized AgNPs are particularly attractive due to their unique properties, potential for deep biofilm penetration, and ability to enhance the efficacy of conventional antibiotics (Piktel et al. 2022).

Given the critical role of biofilm production in the antibiotic resistance and pathogenicity of *A. baumannii* clinical isolates, especially in vulnerable populations such as ICU and burn patients, this study focused on evaluating the anti‐biofilm potential of AgNP@Np and AgNP@Ap. The escalating crisis of antibiotic‐resistant bacteria necessitates the development of novel therapeutic approaches (Hashempour‐Baltork et al. 2019). Nanotechnology facilitates the exploration of antimicrobial agents like plant‐synthesized AgNPs, which offer advantages such as low cost, minimal side effects, and compatibility with bacterial membranes, thereby inhibiting bacterial growth and biofilm formation (Bruna et al. 2021; Mandal et al. 2021).

Numerous studies have documented the effects of plant‐synthesized nanoparticles on bacterial biofilms (Barabadi et al. 2021; LewisOscar et al. 2021; Talank et al. 2022). Among various nanomaterials, silver nanoparticles (AgNPs) have been extensively investigated for their broad‐spectrum antimicrobial activity against bacteria, fungi, and viruses in biomedical contexts (Zhang et al. 2016). Their efficacy against both Gram‐negative and Gram‐positive bacteria is primarily attributed to the induction of oxidative stress and apoptosis (Ansari et al. 2021). While many prior studies have utilized standard laboratory strains, our investigation employed multidrug‐resistant *A. baumannii* (MDR‐AB) clinical isolates with high pathogenicity, enhancing the clinical relevance of our findings.

Silver has a long history of use as an antimicrobial agent, valued for its chemical stability, catalytic properties, and broad‐spectrum activity (Rudrappa et al. 2022; Sim et al. 2018). AgNPs represent a novel class of non‐toxic antibacterial agents that can be synthesized via green, cost‐effective methods. Their ability to target multiple biological pathways simultaneously reduces the likelihood of resistance development (Panpaliya et al. 2019). Thus, AgNPs present a promising alternative to combat escalating microbial resistance.

In this study, the MIC and MBC values for both AgNP@Np and AgNP@Ap against all isolates ranged from 0.1 to 40 μg/mL. Notably, for over 94% of isolates, the MIC and MBC values fell between 0.1 and 3 μg/mL, underscoring the potent growth‐inhibitory activity of these green‐synthesized AgNPs against clinical strains of *A. baumannii*.

Furthermore, we assessed the impact of AgNPs on *bap* gene expression. Our results demonstrate that sub‐MIC concentrations of AgNP@Np (1.171 μg/mL) and AgNP@Ap (1 μg/mL) significantly reduced *bap* gene expression. Additionally, both AgNPs exhibited a strong capacity to inhibit new biofilm formation and disrupt pre‐existing biofilms. The ability of *A. baumannii* to form biofilms is a major clinical concern, as it not only diminishes the efficacy of antimicrobial agents but also contributes to persistent, hard‐to‐treat infections (Römling and Balsalobre 2012). The inhibitory effects of AgNPs on both biofilm formation and associated gene expression observed here suggest a viable strategy to address this challenge. Some research have been done on the effect of nanoparticles synthesized by medicinal plants on biofilm production ability of different bacteria (El‐Naggar et al. 2022; Arya et al. 2018). Our findings align with previous research on the antimicrobial properties of AgNPs. For instance, Hosseini et al. reported an MIC of 128 μg/mL for curcumin nanoparticles against *A. baumannii* (Hosseini et al. 2019). Gharaghie et al. showed that sub‐MIC concentrations of nanoparticles inhibit biofilm formation and reduce *bap* expression (Gharaghie and Shandiz 2018). Rezania et al. found that silver nanoparticles at 6.25 μg/mL inhibited *bap* gene expression in a standard strain and clinical isolates (Rezania et al. 2022). Similarly, Helal F. Hetta et al. reported a 4.5‐fold reduction in *bap* expression after treatment with 25 µg/mL AgNPs (Hetta et al. 2021). Abootalebi et al. synthesized AgNPs using *Ferula asafoetida* extract, reporting MIC and MBC values of 2 μg/mL against clinical *A. baumannii* strains (Abootalebi et al. 2021). The consistency of these results confirms the potential of AgNPs as a viable solution for biofilm‐related infections.

While the present study demonstrates the potent antibacterial and anti‐biofilm activities of the green‐synthesized AgNPs, it is essential to recognize that these nanoparticles are not composed solely of metallic silver. Green synthesis routes invariably produce a hybrid nanostructure comprising a crystalline silver core encased in an organic corona derived from the capping and reducing agents present in the plant extracts. As previously characterized by our group (Ebrahimzadeh et al. 2023), the AgNPs synthesized from *N. pogonosperma* (AgNP@Np) exhibited a spherical morphology with an average crystallite size of 31.68 nm (as determined by XRD using the Scherrer equation), a hydrodynamic diameter of 233 nm (DLS), and a zeta potential of −35.1 mV, indicating good colloidal stability. FT‐IR analysis confirmed the presence of O‐H, C‐H, C = C, and C‐C functional groups on the nanoparticle surface, corresponding to polyphenolic compounds, carbohydrates, and amides derived from the plant extract that serve as capping and stabilizing agents. For AgNP@Ap, while comprehensive nanoparticle characterization is ongoing, the phytochemical profile of *A. persicus* is well‐documented, with essential oils predominantly composed of monoterpenes (α‐pinene, β‐pinene, α‐thujene, sabinene) and sesquiterpenes, and the root extract yielding unique benzodioxole compounds with known biological activities (Bodaghabadi et al. 2021).

The observed disparities in the MIC, MBC, biofilm inhibition, and *bap* gene downregulation between AgNP@Np and AgNP@Ap likely stem from these distinct physicochemical characteristics. The differential phytochemical capping agents—primarily polyphenols and flavonoids from *N. pogonosperma* versus benzodioxoles and terpenoids from *A. persicus*—impart unique surface functionalities, colloidal stability, and biological reactivity. Smaller nanoparticles (such as AgNP@Np) possess a higher surface‐to‐volume ratio, facilitating enhanced bacterial membrane penetration, greater intracellular reactive oxygen species generation, and more efficient interaction with transcriptional machinery, which could potentiate the downregulation of biofilm‐associated genes like *bap*. Conversely, differences in the organic capping layer may modulate the sustained release of bactericidal Ag^+^ ions and the extent of phytochemical‐mediated synergy with the silver core. Critically, the organic residues themselves are not inert; numerous phytochemicals are known to possess intrinsic antibacterial, anti‐adhesion, and quorum‐sensing inhibitory activities. Therefore, the anti‐biofilm efficacy observed here is likely the result of a synergistic effect between the metallic silver core and the bioactive capping layer, rather than the action of silver alone. Overlooking this interplay would lead to an overestimation of the role of pure silver and an underestimation of the contribution of the plant‐derived organic matrix. This nuanced understanding is vital for optimizing synthesis parameters to achieve targeted inhibition of virulence factors in *A. baumannii*.

Green synthesis methods, utilizing plant extracts, provide an environmentally sustainable alternative to conventional chemical synthesis (Santhosh et al. 2022). Despite these promising results, the clinical translation of AgNPs requires further optimization and investigation. Future studies should focus on elucidating their precise mechanisms of action, long‐term safety, potential side effects, and integration into medical devices and treatment protocols. This study provides valuable evidence supporting the potential of green‐synthesized AgNPs for managing biofilm‐associated infections caused by *A. baumannii*. Their inhibitory effects on biofilm formation and virulence gene expression offer a novel approach to combat antimicrobial resistance. Further research is essential to optimize their clinical application, ensure safety, and fully understand their mechanisms of action. The use of green synthesis underscores the importance of sustainable strategies in addressing global antimicrobial challenges.

## 5 Conclusion

This study demonstrates that green‐synthesized silver nanoparticles from *Nepeta pogonosperma* and *Astrodaucus persicus* effectively inhibit biofilm formation and reduce the expression of the biofilm‐associated *bap* gene in clinical isolates of *Acinetobacter baumannii*. The results highlight the potential of these AgNPs as a promising tool to combat biofilm‐related infections. Furthermore, the green synthesis approach provides an eco‐friendly and cost‐effective method for producing AgNPs to address antimicrobial resistance.

In summary, this work offers significant insights into the application of green AgNPs for managing biofilm‐associated infections and underscores the necessity of developing alternative strategies to counter antimicrobial resistance. Future studies should focus on optimizing the clinical use of these nanoparticles and elucidating their detailed mechanisms of action. Such efforts will enhance our understanding of *A. baumannii* pathogenicity and lay the groundwork for innovative therapeutic interventions.

## Author Contributions

**Maedeh Kakavan:** writing – original draft, writing – review and editing; project administration, investigation, methodology, data curation, visualization. **Mehrdad Gholami:** investigation, writing – review and editing, validation, formal analysis, visualization. **Mohammad Ahanjan:** writing – review and editing, visualization, data curation, formal analysis. **Mohammad Ali Ebrahimzadeh:** investigation, writing – review and editing, visualization, methodology, formal analysis, data curation. **Abolfazl Hossein Nataj:** software, data curation, formal analysis, methodology, visualization, writing – review and editing. **Tahoora Mousavi:** visualization, writing – review and editing, formal analysis, methodology. **Hamid Reza Goli:** conceptualization, investigation, funding acquisition, writing – review and editing, visualization, validation, methodology, project administration, supervision.

## Ethics Statement

This study was approved by the Iran National Committee for Ethics in Biomedical Research (National Ethical Code: IR. MAZUMS. REC.1401.283) and was conducted in accordance with the Declaration of Helsinki. Written informed consent was obtained from all patients or their close relatives. All patient data were kept confidential.

## Consent

The authors have nothing to report.

## Conflicts of Interest

The authors declare no conflicts of interest.

## Data Availability

Molecular Microbiology (MMI). The datasets used and/or analyzed during the current study are available from the corresponding author upon reasonable request.
